# The Role of HIV Infection in the Pathophysiology of Gestational Diabetes Mellitus and Hypertensive Disorders of Pregnancy

**DOI:** 10.3389/fcvm.2021.613930

**Published:** 2021-05-12

**Authors:** Wendy N. Phoswa

**Affiliations:** Department of Life and Consumer Sciences, University of South Africa (UNISA), Science Campus, Florida, South Africa

**Keywords:** gestational diabetes, gestational hypertension, highly active antiretroviral therapy, human immune deficiency virus, hypertensive disorders of pregnancy, pre-eclampsia

## Abstract

**Purpose of the Review:** The main objective of this study is to investigate mechanisms associated with gestational diabetes mellitus (GDM) and hypertensive disorders of pregnancy (HDP) in HIV infected pregnant women by looking how placental hormones such as (progesterone and prolactin) and basic haemostatic parameters are regulated in HIV infected pregnancies.

**Recent Findings:** HIV/AIDS are a major global obstetric health burden that lead to increased rate of morbidity and mortality. HIV/AIDS has been associated with the pathophysiology of GDM and HDP. Increased risk of GDM due to highly active antiretroviral therapy (HAART) usage has been reported in HIV infected pregnancies, which causes insulin resistance in both pregnant and non-pregnant individuals. HAART is a medication used for lowering maternal antepartum viral load and pre-exposure and post-exposure prophylaxis of the infant. In pregnant women, HAART induces diabetogenic effect by causing dysregulation of placental hormones such as (progesterone and prolactin) and predispose HIV infected women to GDM. In addition to HIV/AIDS and GDM, Studies have indicated that HIV infection causes haemostatic abnormalities such as hematological disorder, deregulated haematopoiesis process and the coagulation process which results in HDP.

**Summary:** This study will help on improving therapeutic management and understanding of the pathophysiology of GDM and HDP in the absence as well as in the presence of HIV infection by reviewing studies reporting on these mechanism.

## Introduction

Human immune deficiency virus and acquired immunodeficiency syndrome (HIV/AIDS) is the major global health burden affecting ~36.9 million people in the world, including 1.8 million children. “About 25% people don't know their HIV status” (1). The prevalence of HIV infection is higher in low- and middle- income countries, with about 66% living in sub-Saharan Africa. Approximately “19.6 million people are living in East and Southern Africa” (2). In 2017, there were 800,000 new HIV infections in 2017 in the Southern Africa. About 66% adults and 59% children are on anti-retroviral therapy (3).

In the 1980s, the average life expectancy following HIV/AIDS diagnosis was ~1 year. However, with improved medical care; Today, availability of highly active anti retro viral therapy (HAART) started early in the course of HIV infection, have been beneficial in suppressing HIV thus reducing mortality and improving life span of HIV infected individuals (4–6). Unfortunately these drugs lead to development of other adverse health outcomes such as metabolic disorders (Insulin resistance, diabetes mellitus, dyslipidemia, and lipodystrophy) (6, 7). Several studies have reported on the incidence of diabetes mellitus (DM) in HAART experienced individuals (8, 9). The prevalence of DM has been reported to be 10% in HIV individuals receiving medical care, nearly 3.8% higher than in the HIV uninfected population (10, 11).

“Several risk factors such as aging, male sex, obesity, African American, Hispanic, Indian ethnicity, family history, and hepatitis C coinfection, high body mass index (BMI), greater waist circumference, and lower socioeconomic class, have been associated with the development of DM in HIV-infected individuals” (12–18). HIV infection has reported to been linked more with type 2 diabetes mellitus (T2DM) rather than type 1 diabetes mellitus (12). Another type of diabetes that has been widely associated with HIV infection in pregnancy is gestational diabetes mellitus (GDM). Several studies have reported on the incidence of GDM in HIV infected pregnant women. It is speculated that this occurs as a result of HIV infection which causes alterations in the placental hormones which are associated with insulin resistance. Additionally, human immune deficiency virus (HIV) has also been observed as a risk factor for HDP (19). Multiple studies have reported that HIV predisposes pregnant women to HDP (19, 20). Although, the mechanism behind this remains to be ununderstood. It is believed that this occurs as a result of exposure to HAART. HAART “is a treatment given to non-pregnant and pregnant women to prevent viral replication and mother-to-child transmission” (21). This treatment has been reported to alter maternal haemostatic profile (22, 23).

Current literature provides enough evidence that there is a direct link between HIV infection and the pathophysiology of GDM as well HDP. Therefore, more research is required in order to understand association between HIV infection and these diseases. This will help the health care practitioners to improve diagnostic criteria's and care for HIV infected women who are suffering from GDM as well as HDP. And will also help better our knowledge and understanding on the pathophysiology of these conditions in order to improve therapeutic approaches. Hence, the main objective of this study is to investigate mechanisms associated with GDM and HDP in HIV infected pregnant women by looking how placental hormones such as (progesterone and prolactin) and basic haemostatic parameters are regulated in HIV infected pregnancies.

## Gestational Diabetes Mellitus

“Gestational diabetes mellitus (GDM), is defined as excessive glucose intolerance diagnosed during gestation” (24). This disease affects 14% of all pregnancies world-wide (25). The prevalence of GDM in high-income countries is 2–10% and 1.6–14% in low-middle-income countries (26, 27).

Normally, “pregnancy involves altered state of glucose metabolism and insulin sensitivity” (28). However, this change predisposes some pregnant women to GDM (18, 29). Pregnancies affected by GDM are associated with detrimental effects on maternal and fetal health. These effects include, “risk of cesarean and operative vaginal delivery, macrosomia, preterm birth, shoulder dystocia, microangiopathy, neonatal hypoglycemia and hyperbilirubinemia, pregnancy induced hypertension, and pre-eclampsia” (30, 31). Women with a previous history of GDM have increased risk of developing T2DM at an earlier stage in life and cardiovascular diseases (32). Risk factors for GDM include family history of diabetes, advanced maternal age, obesity, ethnicity (no white ethnicity), and previous pregnancy complications (17, 18, 29). “There is currently no cure for GDM, except lifestyle intervention (balanced diet and regular exercise)” (33).

The “prevalence of GDM in HIV uninfected pregnant women is 2–5% of all pregnancies in high income countries” (34, 35) and 2–7% in HIV-infected pregnant women (7, 36–45). “Increased risk of developing GDM in HIV infected women has been reported to be due to the use of HAART which induces insulin resistance in pregnant and non-pregnant individuals” (46). Currently, studies have outlined on the pathophysiology of GDM, however the association between GDM and HIV infection remain obscure.

## Pathophysiology of GDM

Gestational diabetes result from hyperinsulinemia and insulin resistance leading to abnormal glucose intolerance. During the early stages of pregnancy, various metabolic changes occur in the order to promote adipose tissue accumulation. “These changes include increased insulin secretion and insulin sensitivity” (29). Some studies report that insulin sensitivity remain unchanged or decreased during early stages of pregnancy (47, 48). However, “In later stages of pregnancy, insulin sensitivity is reduced due to activation of a number of hormones such as placental lactogen, estrogen, leptin, progesterone, prolactin, cortisol, and adiponectin. It has been reported that insulin resistance plays a central role in the pathophysiology of GDM” (49, 50).

### Placental Induced Insulin Resistance

In the placenta, insulin binds to insulin receptors (IRs) which are present in the placental cells (cytotrophoblasts), thus activating the signaling pathways such as the Ras-extracellular-signal-regulated kinase (Ras-ERK) and the IRS (IR substrate)-PI3K-Akt-mTOR pathway, which are important for placental cellular differentiation, proliferation and metabolism of nutrients (51–53). Insulin is not the only hormone responsible for activating these pathways, growth factors and placental hormones, also play a very important role in their activation (54–56).

During normal pregnancy development the placenta secretes several hormones such as human placental lactogen (hPL), human placental growth hormone (hPGH), progesterone, adiponectin, leptin, prolactin, and cortisol into the maternal blood systems (57). These hormones play a very crucial role in fetoplacental development. However, over secretion as well as under secretion of these hormones has been associated with reduced insulin sensitivity during pregnancy (49, 57, 58).

#### Human Placental Lactogen or Human or Chorionic Somatomammotropin Hormone

Human placental lactogen (hPL) has been shown to “induce insulin secretion from the pancreases during pregnancy” (59). Various studies have documented that hPL can induce insulin resistance (60, 61). Additionally, other studies indicate that hPL can cause peripheral lipolysis insulin resistance (62, 63), although the results are debatable (64). A study conducted by Vasavada et al. (65) in Beta cells of transgenic mice showed that placental lactogen (PL) resulted in over secretion of plasma insulin. Interestingly they also observed increased (2-fold) insulin content in the pancreas. Similar findings were observed by Parsons et al. (66) where they found that placental lactogen secretion resulted in increased islet cell proliferation and insulin secretion. Although, studies have reported on the role of placental lactogen on insulin resistance, However very few studies have reported on human placenta (Table 1). Therefore, more studies looking at the role of human placental lactogen on insulin resistance in different ethnics groups are needed.

**Table 1:** A comprehensive list of studies in this review reporting on hormones associated with GDM.

**Hormone investigated**	**Author**	**Country**	**Cohort type**	**Main findings**
Human placental lactogen (hPL)	(66)	United States	Rat pancreatic cells (islets of Langerhans)	hPL increased insulin secretion.
Human placental lactogen (hPL)	(59)	United States	Rat, mouse, and human pancreatic cells (islets of Langerhans)	hPL induces insulin resistance during pregnancy.
Human placental lactogen (hPL)	(65)	United States	Mice	hPL increased plasma insulin and pancreatic insulin secretion.
Human placental growth hormone (hPGH)	(67)	United States	Mice	hPGH may contribute to the insulin resistance.
Progesterone	(68)	United States	Pregnant women	The use of 17alpha-hydroxyprogesterone caproate (17P) predisposes women to GDM.
Progesterone	(69)	United States	Pregnant women	Women receiving weekly intramuscular 17alpha-hydroxyprogesterone had increased prevalence of GDM.
Progesterone	(70)	Brazil	Rat pancreatic cells (islets of Langerhans)	Progesterone leads to pancreatic cells oxidative stress and may be associated with gestational diabetes in pregnancy.
Progesterone	(71)	United States	Pregnant women	In obese women, the use of 17alpha-hydroxyprogesterone caproate (17P) may increase their chances of developing GDM.
Progesterone	(72)	Iran	Pregnant women	The use of 17alpha-hydroxyprogesterone caproate (17P) may associated with increased risk of GDM in women who conceived *via* assisted reproductive technology.
Adiponectin	(73)	Iran	Pregnant women	Adiponectin is lower in gestational diabetic women.
Leptin	(74)	Austria	Pregnant women	Leptin is correlated with insulin resistance.
Leptin	(75)	Iran	Pregnant women	Leptin is higher in GDM.
Leptin	(76)	China	Pregnant women	Leptin is associated with GDM.
Prolactin	(77)	Australia	Pregnant women	High prolactin is associated with higher glucose and the pathogenesis on GDM.

#### Human Placental Growth Hormone

The human placental growth hormone (hPGH) is found in the placental cells (syncytiotrophoblast), and is said to “replace pituitary growth hormone during pregnancy” (78). This hormone has also been implicated to induce diabetogenic effects result from increased glucose levels and insulin resistance (60, 79). A study conducted by Barbour et al. (68) showed that “hPGH may lead to insulin resistance by increasing the expression of the p85α monomer, which inhibits p85-p110 heterodimer from binding to insulin receptor substrate-1 (IRS-1) protein” (67) thus preventing further insulin signaling leading to substantially reduced glucose uptake (80, 81). “IRS-1 interacts with the p85-p110 heterodimer of phosphatidylinositide 3-kinase (PI3K) which then leads to the activation of this enzyme and subsequent stimulation of adenylate kinase 3 (Akt), resulting in enhanced glucose utilization, and increased glycogen and protein synthesis” (82, 83).

It has been reported that “GDM patients had decreased levels of IRS-1” (49) indicating that in DGM patients there is increased levels of insulin resistance.

#### Progesterone

According to Costrini and Kalkhoff (84) “Progesterone contribute to increased insulin secretion and plasma insulin sensitivity to glucose administration during pregnancy.” Several studies have reported on the diabetogenic effect of progesterone (69–72, 85, 86). Rebarber et al. (87) observed that Administration of progesterone compound; prophylactic intramuscular 17α-Hydroxyprogesterone (17P) resulted in 12.9% incidence of GDM compared to 4.9% control groups. Implying that “17P is associated with an increased risk of GDM”. In contrast a study done by Rosta et al. (88); evaluating the effect of vaginal administration of progesterone on the incident of GDM, showed that there was no significance difference on the incidence of GDM in progesterone treated and the control group. They further concluded that “the use of progesterone is not linked with an increased risk of GDM” (88).

#### Adiponectin

Adiponectin, “is an insulin-inducing hormone secreted by adipose tissue” (89). It is been associated with the pathogenesis of insulin resistance (90). Low levels of adiponectin lead to insulin resistance thus resulting in the pathophysiology of GDM (49, 91).

Studies have shown that the risk of GDM is higher in women with reduced adiponectin levels (92, 93). The general function of adiponectin is to “facilitates insulin action through binding to its receptors AdipoR1 and AdipoR2, thereby leading to induction of adenosine monophosphate dependent kinase (AMPK), PPAR-α” (94).

Adiponectin induces antidiabetic effects by stimulating glucose uptake *via* AMPK by “binding to its receptors AdipoR1 and AdipoR2” (95). “This reduces glucose production in the liver, which can account for the antidiabetic effects of adiponectin” (95). A case control study done by Mohammadi and Paknahad (73) showed decreased serum concentration levels of adiponectin in GDM women indicating adiponectin as a diagnostic tool for detecting the presence of GDM. Although, there are very few studies (96) that have reported on the role of adiponectin on the pathogenesis of insulin resistance in GDM (Table 1), more studies are needed in order to understand how adiponectin is regulated in GDM patients.

#### Leptin

“Is a hormone produced by adipocytes and in low levels by the gastric fundic intestine, placenta, skeletal muscle, and brain” (94, 97). Leptin is important for glucose homeostasis (94). Administration of leptin have been reported to lower insulin secretion (98–102).

A study done *in vitro* showed that “leptin can prevent glucose transporter 2 (GLUT2) phosphorylation, glucose transport, and intracellular adenosine triphosphate (ATP) levels” (98).

Leptin has also been shown to “reduce cyclic adenosine monophosphate (cAMP)-induced insulin secretion, *via* stimulation of phosphodiesterase-3B (PDE3B”) (103–105). Leptin also inhibits protein kinase C (PKC)-induced insulin secretion (106).

The function of leptin is “mediated by the Janus kinase, signal transducer and activator of transcription (JAK-STAT) pathway” (94). “Leptin receptor-mediated JAK–STAT signaling is crucial for monitoring of food ingestion and body weight” (94). Leptin also stimulate PI3K which in turn stimulate IRS-1 followed which activate insulin secretion allowing glucose uptake by the cells (94).

The levels of leptin in diabetes are remain debatable. Several studies report higher plasma leptin levels diabetes mellitus (96, 107, 108). In contrast, Some studies report that reduced levels of leptin correlates with type 1 diabetes (109). However, some studies indicate that there is no correlation between plasma leptin levels and diabetes (110, 111). Interestingly, some research findings report that diabetogenic effect of plasma leptin was only observed in men than women (96, 112, 113).

A study by Kautzky-Willer et al. (74) showed that “high serum leptin levels were correlated with insulin resistance in GDM.” These findings were confirmed by Soheilykhah et al. (75) who observed similar correlation. More interestingly, a study conducted by Yang et al. (76) in Chinese population, investigating the “association between gestational diabetes and plasma leptin levels, leptin G2548, and leptin receptor Gln223Arg polymorphisms” showed that “GDM was only associated with high plasma leptin levels rather that leptin Gln223Arg and leptin receptor polymorphisms.” Although, studies have reported on the association between plasma leptin levels and GDM, however, there are currently no studies that have reported on the association between leptin levels and GDM in the African population (Table 1). More studies are needed in order to see how plasma leptin levels, leptin, and leptin receptor gene polymorphisms are expressed in the African population.

#### Prolactin

Prolactin is a “hormone has also been shown to play role in maintaining glucose homeostasis” (114). Association between prolactin levels and the risk of GDM remain controversial. Some studies report that “excessive prolactin levels have been associated with insulin resistance in diabetes” (115), and some report that “high prolactin levels are associated with decreased risk of diabetes mellitus and impaired glucose tolerance” (116). A prospective study conducted by Wang et al. (117) showed that “high circulating prolactin levels are associated with reduced incident of diabetes mellitus.” In contrast, Daimon et al. (118) showed that “high serum prolactin levels is associated with metabolic effect such as insulin insensitivity in Men.”

Very few studies have associated the levels of prolactin with GDM. High prolactin levels have been linked to the pathogenesis of GDM (77). In contrast, Retnakaran et al. (119) documented that high prolactin levels indicate a sign of lower chances to develop diabetes. Therefore, more studies are needed to confirm how prolactin levels are regulated in GDM.

#### Cortisol

Other factors such as “increased levels of serum cortisol” (120), “Tumor necrosis factor α (TNF α, ILs e.g., IL-6), can interrupt the insulin signaling pathway and can lead to insulin resistance during normal pregnancy” (121).

## Pathophysiology of GDM in HIV Associated Pregnancies

HIV/AIDS has been shown to be the global pandemic that lead both maternal and perinatal morbidity and mortality resulting from pregnancy related complications such as GDM. The placenta is believed to play a primary role in the pathogenesis of gestational diabetes. The placenta is a highly specialized organ that is responsible for normal pregnancy development. It allows feto-maternal exchange of gases and nutrients for fetal development and maintenance to occur. Various pathogens, such as bacterial, fungal, and virus infection (e.g., HIV) can disturb the normal performance of the placenta and lead to pregnancy related complications.

HAART usage during HIV infected pregnancies has been mentioned as the key factor that lead to pregnancy related complications (122–124). HAART-induced GDM is believed to result from the dysregulation of placental hormones during pregnancy. Several studies have reported on how these hormones are regulated in during HIV infection and in the duration of HAART.

Placenta development is regulated by progesterone (125, 126). Reduced levels of progesterone in HIV infection has been reported to disrupt normal pregnancy development (127).

Previous studies have shown that “progesterone levels are lower in HAART treated HIV-infected pregnant women” (128, 129), this explain why there is an increase rate of pregnancy complications in HIV associated pregnancies. A study conducted by Mohammadi et al. (130) on mice revealed that progesterone supplementation to HAART-treated mice improved their placental function. Currently, there are no studies that have reported on the levels of progesterone in HIV associated GDM women. Studies are needed in order to see how this hormone is regulated in HIV induced metabolic disorders.

Leptin is another hormone that has been reported in HIV associated metabolic disorders. This hormone is known for it anti-diabetogenic effects (131). Leptin levels are generally higher in females than in male population (132). In women, leptin levels increase drastically during pregnancy and decrease before or during labor (133).

There is contradictory data on levels of leptin in HIV associated studies. A previous study has reported that leptin levels are decreased in HIV/HAART-associated lipoatrophy (134). In contrast Haffejee et al. (135), observed that high leptin levels in HIV-infected HAART treated women is associated with the pathogenesis of a pregnancy complication (pre-eclampsia). To the best of our knowledge, no studies have reported on the levels of leptin in in HIV infected gestational diabetic women. More studies are needed evaluating the levels of leptin in HIV associated metabolic disorders.

Prolactin is also another hormone that has been found to be elevated during HIV infection (136, 137), but not correlated to HAART usage (138). In contrast, Okeke et al. (139) indicated that prolactin levels are suppressed in HIV infected pregnancy women and that HAART had no effect on the levels of prolactin. No studies have reported on how prolactin in regulated in the presence of HIV infection in GDM women. Therefore, studies are needed in order to understand the pathophysiology underlying GDM during HIV infection.

More interestingly, metabolic disturbance in HIV infected individuals has been correlated with increased serum cortisol. Collazos et al. (140), showed that HIV infected patients who initiated HAART had elevated serum cortisol levels. These findings confirmed the role of cortisol on the metabolic disorders induced by HAART during HIV infection.

In addition, a study evaluating the levels of hPGH showed that HIV status had no effect on the levels of this hormone. These findings were in accordance with the findings by Esemu et al. (141), who investigated the effect of HIV infection on insulin-like growth factor-1 (IGF-1) and angiogenic factors in Cameroonian pregnant women receiving HAART. They reported that “HIV infection did not alter the regulation of both factors” (141). Their findings also confirmed the important of HAART usage in maintaining IGF-1 during pregnancy (141). “IGF-1 is a primary mediator of growth hormone” and placental growth hormone (142). It is also involved in stimulating insulin signaling pathway *via* activation of PI3K-AKT pathway. However, more studies are still needed in order to validate how this hormone is regulated in the presence of HIV infection.

Apart from GDM, HIV infection and its association with increased risk of hypertensive disorders of pregnancy has been reported (19).

## Hypertensive Disorders of Pregnancy

Affect up to 10% of all pregnancies globally (143). The “HDP comprise of gestational hypertension (GH), preeclampsia (PE) and eclampsia (E). A majority of HDP cases are from low and middle income countries” (144). In South Africa, “HDP are the most common direct cause of maternal mortality and account for 18% of all maternal deaths” (145, 146). Hypertensive disorders of pregnancy can also “affect the fetus and the newborn by leading to premature delivery, intrauterine growth retardation (IUGR), abruptio placentae and intrauterine death” (147). “Maternal complications include the HELLP syndrome (hemolyisis, elevated liver enzymes, low platelets), pulmonary edema, acute liver/renal failure, disseminated intravascular coagulopathy, adult respiratory distress syndrome, sepsis, and liver hemorrhage” (144). Despite extensive research on HDP, the pathophysiology of this disorder remains an egnima. Inflammation is one of the commonly reported factors that lead to the development of HDP (148). Among other factors reported to be involved in the pathophysiology of HDP is haemostatic (hematology and coagulation) profile. Studies have demonstrated that “neutrophil to lymphocyte ratios and platelet count may predict disease development, and may help in monitoring disease and the prognosis of HDP” (149–154).

Several risk factors are associated with the pathogenesis HDP. These factors include previous history of PE or GH, multiple pregnancies, polycystic ovarian syndrome, chronic kidney disease, hypertension, diabetes, and autoimmune disorders (155). Recently, HIV has also been observed as a risk factor for HDP (19). Multiple studies have reported that HIV predisposes pregnant women to HDP (19, 20). Although, the mechanism behind this is still to be elucidated. It is believed that this occurs as a result of exposure to highly active antiretroviral therapy (HAART). HAART is a treatment given to non-pregnant and pregnant women to prevent viral replication and mother-to-child transmission (21). This treatment has been reported to induce maternal pro-inflammatory profile, thereby leading to hypertension related disorders of pregnancy (20, 156, 157). Although many studies have focused on the effect of HAART on inflammatory response as the primary cause of HDP in HIV infected individuals. To the best of our knowledge they are very few studies that have reported on the effect of this treatment on the haemostatic profile in association to HDP.

## The Role of Hiv Infection on Haemostatic Profile and Hypertensive Disorders of Pregnancy

HIV has been identified to be strongly linked with haemostatic abnormalities such as hematological disorder, dysregulated haematopoiesis process, and the coagulation process (22, 23). The pathophysiological mechanism behind this link is believed to be through endothelial dysfunction (23, 158). Endothelial dysfunction not only affects haemostatic profile, but is also widely associated with HDP (159, 160).

### Endothelial Dysfunction in Hypertensive Disorders of Pregnancy

“The maternal vascular endothelium is a principal factor involved in the pathogenesis of HDP” (159, 161–165). Under normal conditions there is a balance in the endothelium relaxing and contractile factors that play a pivotal role in regulating arterial compliance, vascular resistance, and blood pressure. However, under abnormal conditions such as HDP, there is an imbalance of these factors. This results to damaged blood vessels which end results leads to endothelial dysfunction which is involved in the pathophysiology of HDP (161–163).

#### Markers of Endothelial Dysfunction Associated With the Pathophysiology of HDP

Several markers of endothelial dysfunction have been associated with HDP (166–168). These markers are “endothelin-1 (ET-1), soluble vascular adhesion molecule and interleukin-8, ELAM-, and endothelial leukocyte adhesion molecule-1. Endothelin-1 is widely reported to be associated with the pathophysiology of PE” (169–172). These studies have shown that “ET-1 is increased in PE” compared to normotensive pregnancies (168, 173–175). Interestingly, it has been reported that “endothelial dysfunction may also lead to diseases associated with maternal liver and brain” (176–178). Severe forms of PE affecting the liver include HELLP syndrome (hemolysis, elevated liver enzymes, low platelet count) (176). A study by Lind et al. (179) indicated that there is correlation between ET-1 and HELLP syndrome in PE. More interestingly, a study of hypertension in an animal model also revealed that there was a strong association between HELLP syndrome and ET-1 activation (180). Eclampsia is another sever form of PE which is accompanied by the presence of seizures (177, 178, 181). The pathophysiology of eclampsia is also reported to involve ET-1, however studies demonstrating this association are lacking. HIV infection has also been identified as a major factor contributing to endothelial dysfunction. Reports on the role of ET-1 on other HDP such as GH are also lacking. Therefore, more studies are needed in order to have a full understanding of the role of ET-1 on the pathophysiology of HDP. Apart from markers associated with endothelial dysfunction in HDP, HIV infection is another identified marker associated with endothelial dysfunction (182, 183).

### The Role of HIV Infection on Endothelial Dysfunction

The role of HIV infection on endothelial dysfunction has been reported to result from “interaction of HIV with host cells that consist of the CD4 receptor, coreceptor chemokines ligand 4 (CXCR4), and chemokines receptor 5 (CCR5)” (184, 185). This interaction occurs with “the help of Glycoprotein 120 (gp120)” (184, 185). This leads to reduced nitric oxide expression followed by endothelial dysfunction (184, 185). Several studies have associated HIV infection with endothelial dysfunction (182, 183). Funderburg et al. (182) reported that “higher plasma HIV RNA levels associates with endothelial dysfunction in HIV-infected patients.” Similarly, “a mouse model expressing HIV viral proteins env, tat, nef, vpu, vpr, and rev demonstrated aortic endothelial dysfunction and increased arterial stiffness” (183). Interestingly, a study by Solages et al. (186), demonstrated that HIV-infected patients had significantly impaired endothelial function, which result from disturbances in their coagulation system as demonstrated by vasoconstriction in comparison to the HIV-uninfected group.

#### Role of HIV Infection on Coagulation

“Endothelial cells produce different molecules that are involved in clotting and fibrinolysis process” (187). These molecules are “Willebrand factor (vWF), tissue plasminogen activator, plasminogen activator inhibitor, and protein S” (187). In HIV-infected individuals, “endothelial abnormality is a common disorder induced by the action of the virus and virus-associated inflammatory response” (188). Such endothelial abnormality “activates the coagulation system, leading to the consumption of coagulation factors” (188). Moreover, it is reported that the levels of vWF are increased in HIV (189). Similar findings have been reported in HDP (190, 191). Therefore, more research on vWF in HIV associated HDP is needed in order to provide possible association between the role of HIV infection in HDP.

## Conclusion

The findings of this review indicate that placental hormones increases insulin resistance during pregnancy however there is very limited data on studies evaluating how these hormones are regulated in HIV infected pregnant women more studies are needed especially from African countries since there is high prevalence of HIV infection as well as high incidences maternal and fetal complication.

## Strength Of the Study

This manuscript highlights on the role of HIV infection in GDM and HDP by looking at hormones associated with GDM and haemostatic parameters associated with HDP in both HIV infected and uninfected pregnancies.

## Limitation of the Study

Clinical or experimental data reporting on the pathophysiology of GDM induced by placental and peripheral hormones in low risk pregnancy vs. HIV infected pregnancy are needed. Also more studies reporting on the haemostatic parameters in HIV infected vs. HIV uninfected pregnancies are needed more especially in Sub Saharan Africa which is a highly affected by HIV infection.

## Author Contributions

WNP conceptualized, wrote, and proof read the manuscript.

## Conflict of Interest

The author declares that the research was conducted in the absence of any commercial or financial relationships that could be construed as a potential conflict of interest.
